# Applying and comparing various nutrient profiling models against the packaged food supply in South Africa

**DOI:** 10.1017/S1368980022000374

**Published:** 2022-08

**Authors:** Tamryn Frank, Shu Wen Ng, Donna R Miles, Elizabeth C Swart

**Affiliations:** 1 School of Public Health, Faculty of Community and Health Sciences, University of the Western Cape, Private Bag X17, Cape Town, Bellville 7535, South Africa; 2 Department of Nutrition, Gillings School of Global Public Health and the Carolina Population Center, The University of North Carolina, Chapel Hill, USA; 3 Carolina Population Center, The University of North Carolina, Chapel Hill, USA; 4 Department of Dietetics and Nutrition, University of the Western Cape, South Africa

**Keywords:** Nutrient profiling, South Africa, Food policy, Nutrients of concern, Obesity

## Abstract

**Objective:**

This study aimed to apply the newly developed Chile Adjusted Model (CAM) nutrient profiling model (NPM) to the food supply in South Africa (SA) and compare its performance against existing NPM as an indication of suitability for use to underpin food policies targeted at discouraging consumption of products high in nutrients associated with poor health.

**Design:**

Cross-sectional analysis of the SA-packaged food supply comparing the CAM to three other NPM: SA Health and Nutrition Claims (SA HNC), Chilean Warning Octagon (CWO) 2019, and Pan-American Health Organisation (PAHO) NPM.

**Setting:**

The SA-packaged food supply based on products stocked by supermarkets in Cape Town, SA.

**Participants:**

Packaged foods and beverages (*n* 6474) available in 2018 were analysed.

**Results:**

Forty-nine per cent of products contained excessive amounts of nutrients of concern (considered non-compliant) according to the criteria of all four models. Only 10·9 % of products were not excessive in any nutrients of concern (considered compliant) according to all NPM evaluated. The CAM had an overall non-compliance level of 73·2 % and was comparable to the CWO 2019 for foods (71·2 % and 71·1 %, respectively). The CAM was the strictest NPM for beverages (80·4 %) due to the criteria of non-sugar sweeteners and free sugars. The SA HNC was the most lenient with non-compliance at 52·9 %. This was largely due to the inclusion of nutrients to encourage, which is a criterion for this NPM.

**Conclusion:**

For the purpose of discouraging products high in nutrients associated with poor health in SA, the CAM is a suitable NPM.

Obesity and non-communicable diseases (e.g. hypertension, diabetes, dyslipidemia and certain cancers) are linked to the consumption of ultra-processed foods high in added sugar, salt, trans- and saturated fats^(1)^. Non-communicable diseases are associated with increased mortality levels, particularly in low- and middle-income countries^(2)^. Changing lifestyles and food systems are synonymous with the nutrition transition, with changing diets shifting away from traditional diets to an increased consumption of ultra-processed, refined foods^(3)^. In sub-Saharan Africa, this nutrition crisis is pronounced, with obesity, and related non-communicable disease prevalence rapidly rising^(4)^. In South Africa (SA), one-third (31 %) of men and two-thirds (68 %) of women have overweight or obesity, and 20 % of women live with severe obesity^(5)^. If the current trend for children continues, 28 % of South African children (aged 5 to 19 years) will have obesity by 2030^(6)^. Similarly, the cost of obesity in SA currently accounts for 1·9 % of the gross domestic product, yet if nothing changes this will increase to 2·6 % by 2060^(7)^.

The double burden of malnutrition (overweight and undernutrition)^(8)^ occurs within an individual over their lifecycle, and across generations within households (stunted/wasted child with an overweight mother). It has long-term consequences for individuals, communities and the economic future of the country^(9)^. Malnutrition in any of its forms leaves one vulnerable to nutritional deficiencies, chronic diseases of lifestyle and infectious diseases including tuberculosis, HIV and coronaviruses^(10,11)^.

Poor nutrition in SA is largely driven by what is available and accessible. Ultra-processed foods high in sugar and fat are cheap sources of energy^(12,13)^. High levels of unemployment and poverty make healthier options unattainable for most^(12)^. Both rural and urban poor communities rely heavily on formal supermarkets and/or both formal and informal fast-food outlets and small shops (spazas) to purchase their food^(14,15)^. Resource constraints drive poor South Africans towards cheap foods resulting in regular consumption of ultra-processed foods^(12,13)^. Multinational food companies account for the majority of the market share^(16)^ of ultra-processed foods. A recent study found that 76 % of assessed packaged foods in SA supermarkets is ultra-processed^(17)^. Consumption habits are continually shifting towards ultra-processed products due to economic, environmental and societal factors such as the price, food type, availability and marketing strategies employed by large corporations^(18)^.

## Uses of nutrient profiling model in South Africa

One way to address the poor nutritional content of ultra-processed products in SA is to implement policies that both disincentivise manufacturers to produce ultra-processed foods and effectively inform consumers about the health risks. Nutrient profiling models (NPM) can assist to achieve this goal. Nutrient profiling is defined as ‘*the science of categorising foods based on their nutritional composition, for reasons related to preventing disease and promoting health*’^(19)^. Well-designed NPM can underpin food and nutrition policies, such as food labelling, child-directed marketing restrictions, taxation and school nutrition standards^(20)^.

In low-to-middle-income countries, the implementation of policies underpinned by NPM has been slow, possibly due to limited resources and a lack of population-level dietary data required to support the development of NPM^(21)^. However, there is a need for stronger, evidence-based policies to promote health and prevent non-communicable diseases in low-to-middle-income countries^(21)^. This is especially true as the World Trade Organisation demands transparent, scientific-based motivations for any country wanting to implement food policies that may restrict trade^(22)^, such as policies aimed at discouraging intakes of products high in nutrients or ingredients associated with poor health^(23)^. Thus, international trade concerns can be minimised by ensuring food policies are based on a transparent and systematic NPM in order to define unhealthy foods^(22)^. Using one NPM across various country-level policies can reduce confusion by ensuring a consistent approach and message to consumers while reducing administrative burden. In SA, a NPM has recently been proposed to identify unhealthy foods and beverages that can be restricted through relevant policies^(17)^.

The current regulations relating to the labelling and advertising of foods in SA, R146, were implemented in 2010^(24)^. According to R146, it is mandatory to include an ingredient list on packaged food labels, but a nutrition information panel (NIP) is optional^(24)^. An updated draft of these regulations, R429 of 2014^(25)^, exists but has not been promulgated. This draft R429 recommended a mandatory NIP to promote transparency of the nutritional content of the foodstuff and to verify compliance to nutrient profiling recommendations for health and nutrition claims. Moreover, trans-fats regulations prohibiting more than 2 g of trans-fat per 100 g of oil or fat were implemented in 2011^(26)^, and SA implemented mandatory Na limits for various processed food categories in June 2016^(27)^. The SA National Department of Health has been working to finalise R429, with the intention to include a NPM that is suitable for the SA context and discourages the supply and demand of ultra-processed foods and beverages containing high amount of nutrients or ingredients linked to poor health outcomes. Additionally, they have expressed interest in food policies, such as front-of-package warning labels^(28)^.

This study aimed to apply a newly developed NPM to the packaged food supply in SA and compare its performance to other existing NPM as an indication of suitability for use, given the SA Department of Health’s interest in it.

## Methods

### Models selected for comparison

A rigorous process has previously been followed to identify a NPM suitable for use in food policy in SA^(17)^. This newly developed NPM is referred to as the Chile Adjusted Model (CAM) in this paper. Its performance that needed to be tested alongside existing NPM developed for similar purposes. The models chosen for the comparison included those that have some resonance with the food policies under consideration. These include the Chilean Warning Octagons (CWO) which Chile has successfully used to implement a comprehensive package of food policies^(29,30)^, and the Pan-American Health Organisation (PAHO) model, as the first proponent of restrictive food policies^(31)^. It was also appropriate to include the existing NPM in SA^(32)^ in the assessment.

NPM details are summarised in Table 1. Briefly, the NPM^(32)^ adopted from Food Standards Australia and New Zealand Food Standards Australia/New Zealand’s (FSANZ) NPM (which in turn was adapted from the UK Ofcom NPM)^(33)^ is currently used as the basis for assessment of health and nutrition claims in SA’s draft R429^(25)^ and referred to as the SA Health and Nutrition Claims (SA HNC) in this paper. It has also been validated in SA for the purpose of underpinning marketing restrictions to children^(32)^. The Centre of Excellence for Nutrition at North West University proposed the SA HNC^(32)^ which was then incorporated into the draft R429 in 2014 by the SA Department of Health. The NPM referred to as the CWO^(29)^ was developed by the Chile Ministry of Health to underpin policy related to warning front-of-package labelling (FOPL), restriction of marketing to children and regulation in the school environment. Promulgated in 2012, the CWO was implemented in three phases: 2016, 2018, and 2019. The CWO has gained attention for its success in Chile^(30)^ and thus is included in this study applying the most stringent phase, the CWO 2019, as it contains the final cut points that the regulation achieved. The PAHO model was published in 2016 and developed through rigorous work by an expert consultation group composed of recognised authorities from Latin America in the field of nutrition. Its purpose is to identify processed foods excessive in nutrients of concern that can be used to construct food policy^(31)^, as seen in Mexico’s mandatory FOPL^(34)^. The fourth model, the CAM, acknowledges the success of the CWO^(30,35,36)^ but was adjusted by the authors to replace added sugar with free sugar in its qualifying criteria of ingredients, include presence of non-sugar sweetener (NSS) criteria and exclude the energy criteria. The reason for the inclusion of free sugar as opposed to added sugar as a qualifying ingredient in which total sugar values are then assessed is that 100 % fruit juice is excluded from PAHO and CWO 2019. Recent literature suggests that excessive sugar consumption from 100 % fruit juice is harmful and should be limited^(37,38)^. Likewise, replacement of sugar with NSS should be restricted given the association of the latter with increased morbidity^(39,40)^. The inclusion of NSS is similar to PAHO^(31)^ and Mexico’s^(34)^ recently introduced NPM. Energy was excluded during the NPM development process as only 2·3 % of products evaluated were exclusively high in energy, but not any other nutrient (described elsewhere in detail)^(17)^.

**Table 1:**
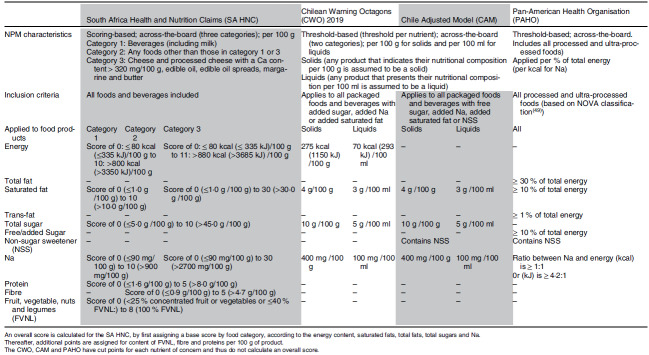
Characteristics of four nutrient profiling models (NPM)

Currently, there is no gold standard for classifying the healthfulness of foods to use for NPM validation. The current study developed algorithms to apply four NPM to a cross-sectional analysis of the SA-packaged food supply collected in 2018. The purpose is to show how similarly or differently the same set of products available in SA would be considered as compliant or not under these four NPM.

### Sampling procedures

Nutritional information of packaged food and beverages was collected between February and March 2018, in six supermarket chains that accounted for more than 50 % of the grocery retailer market share in SA in 2018^(41)^. Selection of these stores ensured a representative sample of packaged foods available on the SA market. Data collection was conducted in Cape Town in the middle-income suburb Durbanville (at Pick ‘n Pay, Woolworths, Checkers and Spar), as well as in the low-income suburbs of Langa (at Shoprite) and Khayelitsha (at Boxer and Pick ‘n Pay). Fieldworkers took photographs of all packaged food products in the store at the time of data collection. Photographs captured all sides of food containers and include all information from the product packaging (e.g. product name, package size, bar code, ingredients and NIP).

### Fieldwork and data entry

Trained university graduate fieldworkers followed a standardised protocol developed by The George Institute (TGI) to capture and submit photographs of food labels to the Foodswitch database using cellphone cameras. TGI supervised a team of data capturers to view the photographs and enter product information into the Foodswitch database using standardised methods and quality control checks.

**Fig. 1:**
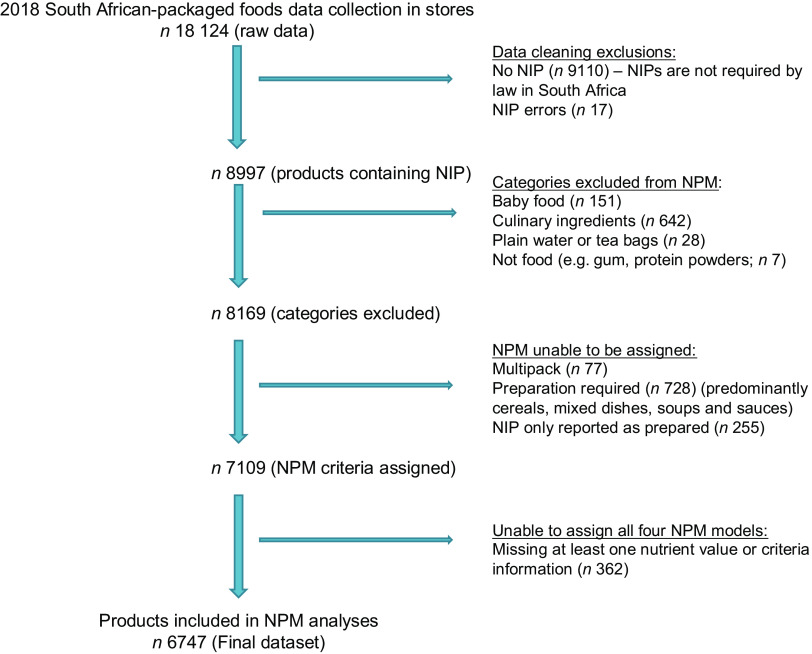
Flow diagram representing initial, and final dataset, and reasons for exclusion. NIP, nutrition information panel; NPM, nutrient profiling model

Products are classified into eleven food categories and four beverage categories. Conversion of foods and beverages requiring reconstitution (e.g. liquid concentrate beverages) from an ‘as sold’ form to an ‘as consumed’ form was based on information retrieved from product photographs when available. Data collection comprised of 18 124 products, of which 6747 had sufficient information for NPM analyses. Figure 1 provides a flowchart of sample sizes. Data cleaning and analyses were performed using STATA (version 15, StataCorp.). The nutrient content of products in the database was verified by identifying outliers and cross-checking against the original photographs of each product and corrected when possible.

Table 2 represents the final number of products in various food groups included in the dataset (*n* 6747). Most (78·4 %, *n* 5290) are foods and 21·6 % (*n* 1457) are beverages.

**Table 2:**
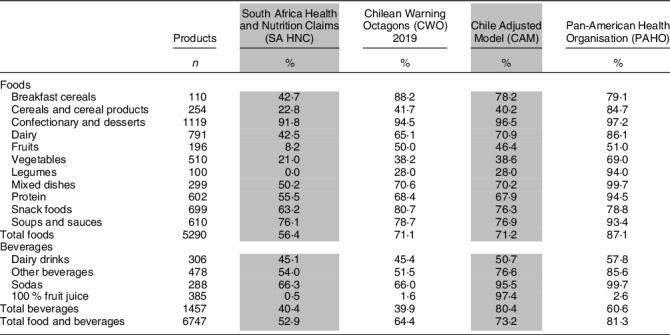
Proportion of South African-packaged foods and beverages that are non-compliant per NPM overall, for foods and beverages, and by select categories

### Testing selected nutrient profiling models

Products were excluded from NPM analyses if missing information that hindered scoring for any of the four NPM. The SA HNC requires calculations of a fruit, vegetable, nuts and legumes (FVNL) score based on the percentage of fruits and vegetables contained in a product. FVLN scores were calculated based on the percentage of FVLN in ingredient lists when reported and manually estimated for products without this information (out of 957 products 62 % were manually estimated). A similar methodology for calculation was followed as described by Bernstein *et al*.^(42)^. First, a FVNL score of 0 was assigned to subcategories without any FVNL (e.g. fats and oils). For groups where products might contain FVNL, each product was individually reviewed and the order of ingredients, number of FVNL ingredients compared to number of non-FVNL ingredients, form of the FVNL ingredients (concentrated or non-concentrated), and type of product were taken into consideration when assigning points. Likewise, if free sugar values were not available but added sugar was listed, then the free sugar content was estimated according to the method proposed by PAHO^(31)^. A registered dietitian assigned all classifications.

Products were determined to be either compliant or non-compliant based on the nutritional criteria of each NPM. For the SA HNC, compliance includes products meeting criteria for carrying a health claim: for beverages a score of less than 1; processed cheese and fats a score of less than 28; and other foods a score of less than 4. For the CWO 2019, products excluded from carrying a warning FOPL are considered compliant (i.e. nutrients did not exceed criteria for energy, sugar, Na or saturated fat). For PAHO, products meeting all the stipulated criteria for total fat, saturated fat, trans-fat, Na, free sugar and NSS are considered compliant. Likewise, products under the CAM are compliant when not exceeding thresholds for sugar, saturated fat, Na or containing any NSS.

### Data analysis

The four NPM were compared by the number and proportion of foods classified as either compliant or non-compliant, overall and by food category. Differences across models regarding the proportion and mean number of foods identified as non-compliant were explored by using tests of proportions and *t*-tests, respectively. The mean contents of nutrients of concern among non-compliant products were calculated and compared across NPM. The level of agreement between each NPM was evaluated using pairwise correlation coefficients. A *P*-value of <0·05 was used to determine a level of significance.

## Results

### Numbers and proportions: results of various nutrient profiling model

Table 2 presents the percentage of products non-compliant for each NPM for foods, beverages and overall, as well as by category. The SA-packaged food supply had the highest non-compliance rate by the PAHO (81·3 %, *n* 5488). For foods, the non-compliance levels were similar for CAM and the CWO 2019 (71·2 %, *n* 3766 and 71·1 %, *n* 3763, respectively). However, the CAM had the highest level of non-compliance for beverages (80·4 %) due to the criteria of free sugars and NSS. The most lenient model was the SA HNC with a non-compliance level of 52·9 % (*n* 3570). This was largely due to a lower share of food products considered non-compliant (56·4 %) than the other NPM, although the beverage share was comparable to CWO 2019. Within seven product categories (legumes, fruits, vegetables, cereal products, diary, breakfast cereals and mixed dishes), the SA HNC was more lenient than any other NPM, by at least 15 percentage points. Conversely, the PAHO was at least 15 percentage points more non-compliant than any other NPM for seven food categories (mixed dishes, protein, legumes, soups and sauces, dairy, cereal products and vegetables). Although the CWO 2019 and CAM had similar results for food categories, one category, breakfast cereals, had noticeably more (10·0 %, *n* 11) non-compliant products for the CWO 2019. Among these products, all eleven were high in energy but did not exceed the CWO 2019 compliance level for Na, sugar or saturated fat. The discrepancy was due to the energy criteria for CWO 2019 omitted in CAM.

For beverages, the CAM had twice as many non-compliant products as both the SA HNC and CWO 2019 (80·4 % non-compliant *v*. 40·4 % and 39·9 %, respectively), and 20 percentage points higher non-compliance than the PAHO (non-compliance level of 60·6 %). The CAM was at least 22 percentage points more non-compliant than the CWO 2019 and the SA HNC for sodas, 100 % fruit juice and other beverages. Although CAM was similar to the PAHO for sodas, the PAHO had more non-compliant products in dairy drinks and other beverages categories (7·12 and 8·99 percentage points more, respectively). Most of these products (*n* 65) were low in energy, but high in Na (*n* 21), free sugar (*n* 17) and/or total fat (*n* 22). The category with the largest difference overall was 100 % fruit juice due to the free sugar qualifying criteria of the CAM. The PAHO, SA HNC and CWO 2019 had a non-compliance rate of 2·6 % or less, whereas the CAM non-compliance rate was 97·4 % (*n* 375).

These findings align with the test of proportions where the difference in the percentage of non-compliant products was largest between the SA HNC and the PAHO models, and smallest between the CAM and PAHO models. For foods, specifically there was virtually no difference between the CAM and CWO 2019 (Appendix 1).

**Fig. 2:**
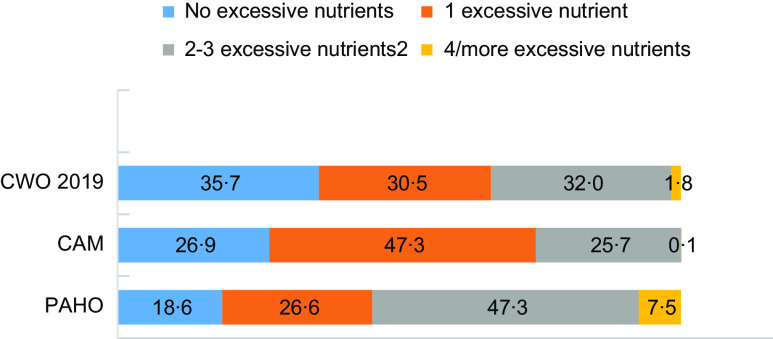
Total proportion of products with 1, 2–3 or 4 and more ‘excess nutrients’ – PAHO, CWO 2019 and CAM NPM. NPM, nutrient profiling model; CWO 2019, Chilean Warning Octagon; CAM, Chile Adjusted Model; PAHO, Pan-American Health Organisation

As the SA HNC includes both nutrients to encourage and limit, it was excluded from analyses that considered nutrients in excess exclusively. Unlike the three other models that provide threshold-based scores, the SA HNC provides a cumulative score, and thus the SA HNC cannot be directly compared to the other three NPM only regarding excessive nutrients. Figure 2 indicates excessive nutrients by number for the PAHO, CAM and CWO 2019. The PAHO model contains the largest number of products with four or more excessive nutrients (e.g. Na, free sugar, saturated fat, trans-fat, total fat and/or NSS), whilst the CAM is most likely to have only one nutrient in excess. Despite this, overall, the CAM still has more products excessive in at least one nutrient when compared to the CWO 2019. The PAHO model has the largest number of excessive products overall.

### Level of agreement in compliance of different nutrient profiling model when assessing the South African-packaged food supply, overall and by category

Table 3 presents details on the level of agreement in compliance of different NPM overall and by category. Appendix 2 provides a comparison across NPM of the differences in the mean number of products with excess nutrients by category. Forty-nine per cent of all products (*n* 3331) contained excessive amounts of nutrients of concern and were non-compliant according to all four NPM assessed. Just over half of all foods (52·7 %; *n* 2788) and one-third of all beverages (37·3 %; *n* 5430) were classified as non-compliant. Categories in which more than half the products were non-compliant according to all NPM included confectionary and desserts, soups and sauces, sodas and snack foods. The PAHO model had several categories with higher exclusive non-compliance than the other models. At least 30 % of cereal products, legumes and vegetables were non-compliant only under the PAHO model due to excessive amounts of Na. Of the products non-compliant only to the PAHO, 95·5 % of legumes (*n* 63), 87·6 % of vegetables (*n* 134) and 99·0 % of cereal products (*n* 103) were high in Na. CAM is the only NPM that has a category (100 % fruit juice) with 95 % greater non-compliance. The only products in this category that are CAM compliant are coconut water and lemon juice. All other 100 % fruit juice products exceeded the sugar threshold according to the CAM criteria.

**Table 3:**
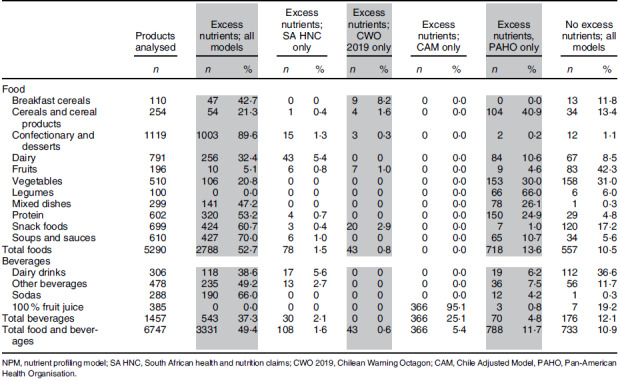
Level of agreement in compliance of different NPM when assessing the SA-packaged food supply, overall and by category

Only 10·9 % (*n* 733) of all products were not excessive in any nutrients of concern according to the four NPM. Compliant products were mainly from dairy drinks (36·6 %, *n* 112), fruits (42·3 %, *n* 83) and vegetables (31·0 %, *n* 158). Snack foods found compliant for all four models (17·2 %) consisted of products such as plain nuts and seeds, plain popcorn, plain rice and corn cakes, crisp bread and some nut butters.

None of the NPM are completely aligned (pairwise correlation coefficients, Table 4; and level of agreement, Appendix 3). The CAM and CWO 2019 were most closely aligned overall, for food, for any excess (0·75 and 0·92) and number (0·84 and 0·91) of excess nutrients. However, there was poor alignment between the CAM and other NPM for beverages, with the highest alignment for beverages between PAHO and CWO 2019 (at 0·66 for any nutrient in excess). As explained previously, the SA HNC was not included in evaluations of nutrients in excess.

**Table 4:** Pairwise correlation coefficients between NPM and any or specific number of nutrients

		Pairwise correlation coefficients between CWO 2019, CAM and PAHO for any nutrient in excess		Pairwise correlation coefficients between CWO 2019, CAM and PAHO for number of nutrients in excess	
		CAM	PAHO	CAM	PAHO
CWO 2019	Food	0·9176	0·5433	0·9089	0·4322
	Beverages	0·3919	0·6566	0·5174	0·4292
	All	0·7505	0·6043	0·8351	0·4813
CAM	Food	–	0·5988	–	0·5291
	Beverages	–	0·3300	–	0·4362
	All	–	0·4699	–	0·5058

NPM, nutrient profiling model; CWO 2019, Chilean Warning Octagon; CAM, Chile Adjusted Model; PAHO, Pan-American Health Organisation.

SA Health and Nutrition Claims NPM not included in this comparison due to the different types of model.

### Comparison of nutrients of concern between nutrient profiling model

In order to compare how effectively the various NPM cut points achieved the desired outcome for the nutrients of concern, means by compliance and non-compliance were examined (see Table 5).

**Table 5:**
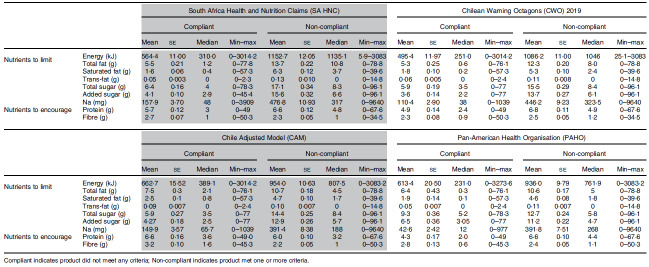
Mean content of nutrients of concern per 100 g of product by compliance to NPM criteria

For all NPM, mean Na content was below 160 mg/100 g among compliant products. The SA HNC had the highest mean Na content in compliant products (157·9 mg/100 g) and the PAHO model the lowest at 42·6 g/100 g. Non-compliant products Na mean ranged from 391·8 mg/100 g (PAHO) to 476·8 mg/100 g (SA HNC). The highest mean saturated fat content in the compliant group was 2·5 g/100 g, for the CAM NPM. For both total sugar and added sugar, PAHO had the highest compliant content (9·3 g/100 g and 6·5 g/100 g, respectively), while the CWO 2019 had the lowest (5·9 g/100 g and 3·6 g/100 g, respectively).

Mean energy was below 630 kJ/100 g in all four NPM for compliant products. The CAM had a higher mean than the CWO 2019 for energy (663 kJ/100 g and 495 kJ/100 g, respectively). The PAHO has a lower compliant mean (613 kJ/100 g) than the CAM. The CWO 2019, the only model to include energy as a nutrient of concern, had 2123 observations for ‘high energy’. Only 91 (1·4 % of the total sample) of these observations were compliant according to the CAM (due to most energy-dense products containing excessive amounts of other nutrients of concern). Interestingly, although protein and fibre are promoted by the SA HNC model, it was the CAM that had the highest averages of these nutrients in the compliant group (6·6 g/100 g and 3·2 g/100 g, respectively).

Note that NSS and FVNL were not included in this nutrient-level analysis. NSS is currently not included on the NIP of packaged foods in SA and although the presence of NNS could be identified via the ingredient list, the amount of NNS could not be compared. The FVNL score was not included in this assessment, as the calculated amount was an estimate and would be inaccurate to compare across different NPM.

## Discussion

According to the criteria of the four NPM assessed, between half and 80 % of all products assessed contained excessive amounts of nutrients of concern and are considered non-compliant. This affirms like in many other countries^(9)^ that SA’s nutrition transition is advanced^(8)^, and the packaged food supply includes predominately ultra-processed foods in excess of nutrients of concern and may be considered unhealthy^(17)^. Categories especially high in non-compliant products were confectionary and desserts, soups and sauces, sodas, and snack foods. Only 11 % of products were found to be compliant according to all the NPM analysed and comprised of products beneficial to health, such as fruits and vegetables, and healthier snacks like plain nuts and seeds and low/no sugar dairy drinks.

Similar to other studies, the PAHO model had the highest level of non-compliance^(22)^. Less than 20 % of the current SA-packaged food supply would be exempt from a warning FOPL (an example of a food policy) should the PAHO be used for this purpose. Conversely, the most lenient model was the SA HNC, which found almost half (47 %) of the products compliant. Its original intended use was to allow health claims, and it is the only NPM assessed to include both nutrients to limit and encourage^(25)^. This difference was particularly evident in the legumes, fruits, vegetables, cereal and cereal products, dairy, breakfast cereals and mixed dishes categories, where non-compliance levels were at least 15 % lower than the other NPM. In all of these categories, it is easy to score positive points for fibre, protein and/or FVNL as these categories of food often contain these ingredients. There has been some criticism that NPM that contain nutrients to encourage do not achieve the goal of promoting whole-grain and whole foods due to their focus on energy density rather than nutrient density^(43)^. Unfortunately, the addition of these nutrients to encourage does not automatically cancel out the negative health consequences of consuming large amounts of nutrients of concern. This supports apprehensions that the addition of nutrients to encourage in a NPM can confuse the matter when trying to identify unhealthy foods to restrict in food policy^(22)^. In fact, the mean fibre and protein content of products compliant with the CAM was higher than the SA HNC; thus, a focus on restricting nutrients of concern does not necessarily negatively bias against healthier products.

The CAM and CWO 2019 had similar levels of non-compliant foods, but the CAM was stricter for beverages. This is due to the additional criteria for NSS, as well as the qualifying inclusion criteria of free sugar instead of added sugar in the CAM. This criterion ensures that high sugar 100 % fruit juices^(37,38)^ that contain fruit concentrate are not inadvertently excluded from being identified as non-compliant in the NPM. As the PAHO assesses free sugar rather than total sugar, one may expect the high free sugar content of 100 % fruit juice to be flagged as non-compliant by the PAHO model. However, the processing level qualifying criterion of the PAHO model exempts 100 % fruit juice as it is not considered processed^(44)^.

The CAM which does not include a criterion for energy had a similar mean energy content to the CWO 2019 which does include a threshold for total energy for compliant products. The mean saturated fat and trans-fat values are slightly higher for the CAM than the other models, which is likely due to the exclusion of an energy criteria for this NPM. However, although the CAM has the highest mean for saturated fat in the compliant group, it is still well below the cut point for foods (4 g) and beverages (3 g). Similarly, the mean trans-fat content in the compliant group is well below the cut point provided in the SA trans-fat regulation^(26)^.

Based on the results of the current study, the CAM is an appropriate NPM for its intended purpose. Out of the four NPM, the CWO 2019 and CAM were most closely aligned to each other. As the CAM was adapted from the CWO 2019, this is to be expected. The difference in alignment for beverages specifically indicates that the CAM’s adaptations for ingredient criteria of free sugar and NSS had the intended outcome. Despite CAM having a lower number of products excessive in more than one nutrient of concern in comparison with the CWO 2019 and PAHO, this should not negatively affect its usage in policy as overall it had the second highest level on non-compliant products, and usage in policy is intended to be binary, based on the overall non-compliance of at least one nutrient profiling criterion and not the sum total of the number of excessive nutrients within one product.

The PAHO model may be considered too strict to practically use in policy. There is plenty of evidence suggesting the level of processing as addressed by the PAHO approach is one of its strengths given growing concern and evidence around the role of ultra-processing as an independent factor beyond that of nutrients on poor health outcomes^(1)^. However, with so few compliant products, particularly in the categories of legumes, vegetables and cereal products where it was much stricter than the other models, the public may become indifferent to its presence should it be used in policy as there will be very few viable compliant options, although this could encourage reformulation by manufacturers. It is the only assessed NPM to evaluate the quantity of free sugar rather than total sugar. From a health standpoint, free sugar is more appropriate to assess than total sugar; however from a regulatory standpoint, there is no way to differentiate between free and total sugar^(45)^ making monitoring and evaluation of free sugar content extremely difficult without access to recipes which are often protected by companies. This is one of the reasons why most NPM used in regulation assess thresholds of total sugar rather than free sugar^(46)^.

Several concerns arise around the SA HNC model. Firstly, calculating the FVNL score is not practical in the SA context. Without regulation requiring reporting of these values, rough estimations have to be made^(33,42)^, making monitoring and evaluation challenging and creating difficulty in identifying dishonest manufacturers who may manipulate values. This is not aligned with recommended good policy objectives^(46)^. The points awarded for nutrients to encourage inadvertently diminish its effectivity at identifying nutrients to limit, as can be seen in the lower level of non-compliant products in this NPM. This model is currently recommended in SA’s draft regulation R429 to identify products permitted to carry a health or nutrition claim rather than to identify harmful nutrients of concern. As such, it may still have a role to play in policy specifically for health claims as a subsequent step to the CAM. It is important that products do not carry both a warning for excessive nutrients of concern and a health claim encouraging consumption of certain healthy components as this has been found to create mixed messages on the healthfulness of foods and confuse consumers^(47)^. In other words, provided a product is first classified as not excessive in nutrients of concern according to the CAM criteria, a health claim could be allowed for products that also meet the SA HNC criteria.

### Limitations and assumptions

Although data were collected in large supermarkets in the Western Cape with the intent of capturing a representative sample of packaged foods available on the SA market, it is possible that certain products only occur in certain shops or geographical areas not included in this data collection. Additionally, products were only included in the study if a NIP was present. As NIP are not currently a legal requirement in SA, many products had to be excluded from NPM analyses. It is recommended that the SA government enforce mandatory regulations for a NIP on all packaged foods. The information this panel provides can be used to assess compliance with various food regulations. The NIP should be transparent, standardised and easy to interpret as aligned with Codex guidelines^(48)^.

Certain assumptions were made to compare across the different NPM. All products were treated equally, and consumption frequency as part of usual dietary intake was not considered. Products were included if they could be assessed according to the inclusion criteria for all four NPM. In real-life settings, some items are included by one NPM and excluded from analysis by another NPM. These items were not included in this analysis. Likewise, as free sugar and FVNL values were not available, assumptions made may have not always been correct. As the score-based SA HNC model includes points for both nutrients to limit and encourage and the threshold-based PAHO, CWO and CAM only include thresholds regarding nutrients of concern, it was not possible to compare across all four models specifically for excessive nutrients of concern.

## Conclusion

Based on the assessment of four NPM against the SA-packaged food supply, the CAM is a suitable NPM to underpin food policies in SA. It is able to identify unhealthy products high in saturated fat, sugar, Na or containing NSS. Policies it can support include those that require the identification of unhealthy foods to be regulated, such as for the restriction of marketing to children, regulation in the school food environment and for warning FOPL.

## Supplementary material

For supplementary material accompanying this paper visit https://doi.org/10.1017/S1368980022000374. click here to view supplementary material
