# Supplementary material for: Applying and comparing various nutrient profiling models against the packaged food supply in South Africa
Source: Public Health Nutr. 2022 Feb 16;25(8):2296–307. doi: 10.1017/S1368980022000374 (PMC9378746; doi:10.1017/S1368980022000374)
Supplement: Supplementary file 1 [file S1368980022000374sup.zip › S1368980022000374sup001.docx]

Appendix 1. Test of proportions of South African pre-packaged foods and beverages that are non-compliant per nutrient profiling model overall; for foods and beverages; and by select categories (using the CAM and SA HNC nutrient profiling models as reference models)

|  | Difference in % non-compliant (CAM NPM as reference) | | | Difference in % non-compliant (SA HNC NPM as reference) | |
| --- | --- | --- | --- | --- | --- |
|  | CAM% -SA HNC% | CAM% -CWO% | CAM%-PAHO% | SA HNC% -CWO% | SA HNC% -PAHO% |
| **FOODS** | | | | | |
| 1. Breakfast cereals | 35.45** | -10.00* | -0.91 | -45.46** | -36.36** |
| 2. Cereals & cereal products | 17.32** | -1.58 | -44.49** | -18.90** | -61.81** |
| 3. Confectionary & Desserts | 4.74** | 2.06* | -0.72 | -.02.68* | -5.45** |
| 4. Dairy | 28.43** | 5.82* | -15.17** | -22.63** | -43.62** |
| 5. Fruits | 38.27** | -3.57 | -4.59 | -41.84** | -42.86** |
| 6. Vegetables | 17.65** | 0.39 | -30.39** | -17.25** | -48.04** |
| 7. Legumes | 28.00** | 0.00 | -66.00** | -28.00** | -94.00** |
| 8. Mixed dishes | 20.07** | -0.33 | -29.43** | -20.40** | -49.50** |
| 9. Protein | 12.46** | -0.50 | -26.58** | -12.96** | -39.04** |
| 10. Snack foods | 13.02** | -4.44* | -2.58 | -17.45** | -15.59** |
| 11. Soups & sauces | 0.82 | -1.8 | -16.56** | -2.62 | -17.38** |
| **Total All Foods** | **14.84**** | **0.06** | **-15.86**** | **-14.78**** | **-30.70**** |
| **BEVERAGES** | | | | | |
| 12. Dairy drinks | 5.56 | 5.23 | -7.19 | -0.33 | -12.75** |
| 13. Other beverages | 22.59** | 25.10** | -9.00** | 2.51 | -31.59** |
| 14. Sodas | 29.17** | 29.51** | -4.17** | 0.35 | -33.33** |
| 15. 100% Juice | 96.88** | 95.84** | 94.81** | -1.04 | -2.08* |
| **Total All Beverages** | **39.95**** | **40.50**** | **19.77**** | **0.549** | **-20.19**** |
| **TOTAL FOOD & BEVS** | **20.26**** | **8.79**** | **-8.17**** | **-11.47**** | **-28.43**** |

NPM – Nutrient profiling model; CAM – Chile adjusted model; SA HNC – South African health and nutrition claims; CWO – Chile warning octagon 2019; PAHO – Pan American Health Organization

*p<0.05 **p<0.01

Appendix 2. Comparison in mean number of products with “excess nutrients” of South African pre-packaged foods and beverages that are non-compliant per nutrient profiling model overall; for foods and beverages; and by select categories (using ttest in Stata)

|  | **Difference in mean number of products with “excess nutrients”** | | |
| --- | --- | --- | --- |
|  | **CAM NPM as reference** | | **CWO 2019 NPM as reference** |
|  | **CAM – CWO 2019** | **CAM - PAHO** | **CWO 2019 - PAHO** |
| **FOODS** | | | |
| 1. Breakfast cereals | -0.856** | -0.300** | 0.555** |
| 2. Cereals & cereal products | -0.154** | -1.075** | -0.921** |
| 3. Confectionary & Desserts | -0.688** | -0.517** | 0.172** |
| 4. Dairy | -0.005 | -1.235** | -1.230** |
| 5. Fruits | -1.020** | -0.061** | 0.041 |
| 6. Vegetables | -0.008 | -0.982** | -0.975** |
| 7. Legumes | -0.030 | -1.010** | -0.980** |
| 8. Mixed dishes | -0.087** | -1.759** | -1.672** |
| 9. Protein | -0.116** | -1.515** | -1.399** |
| 10. Snack foods | -0.707** | -0.425** | 0.282** |
| 11. Soups & sauces | -0.343** | -1.200** | -0.857** |
| **Total All Foods** | **-0.328**** | **-0.934**** | **-0.606**** |
| **BEVERAGES** | | | |
| 12. Dairy drinks | 0.062 | -0.605** | -0.667** |
| 13. Other beverages | 0.439** | -0.494** | -0.933** |
| 14. Sodas | 0.549** | -0.528** | -1.076** |
| 15. 100% Juice | 0.958** | 0.935** | -0.023* |
| **Total All Beverages** | **0.519**** | **-0.146**** | **-0.665**** |
| **TOTAL FOOD & BEVERAGES** | **-0.145**** | **-0.764**** | **-0.619**** |

NPM – Nutrient profiling model; CAM – Chile adjusted model; CWO 2019 – Chile warning octagon 2019; PAHO – Pan American Health Organization

*p<0.05 **p<0.01

Appendix 3. Pairwise k values for the four nutrient profiling models

|  |  | | **CAM** | | **SA HNC** | | **PAHO** | |
| --- | --- | --- | --- | --- | --- | --- | --- | --- |
| **CWO 2019** | **Food** | | 0.9176 (Almost perfect) | | 0.5517 (Moderate) | | 0.4814 (Moderate) | |
|  | **Beverages** | | 0.2715 (Fair) | | 0.8829 (Almost perfect) | | 0.6025 (Moderate) | |
|  | **All** | | 0.7349 (Substantial) | | 0.6370 (Substantial) | | 0.5501 (Moderate) | |
| **CAM** | **Food** | | - | | 0.5417 (Moderate) | | 0.5310 (Moderate) | |
|  | **Beverages** | | - | | 0.1909 (Slight) | | 0.2941 (Fair) | |
|  | **All** | | - | | 0.4501 (Moderate) | | 0.4573 (Moderate) | |
| **HNC** | **Food** | | - | | - | | 0.2538 (Fair) | |
|  | **Beverages** | | - | | - | | 0.5278 (Substantial) | |
|  | **All** | | - | | - | | 0.3398 (Fair) | |
| Level of agreement using the Kappa statistic | | | | | | | | |
| Slight:  0.0-0.20 | | Fair:  0.21-0.40 | | Moderate:  0.41-0.60 | | Substantial:  0.61-0.80 | | Almost perfect:  0.81-0.99 |

CAM – Chile adjusted model; SA HNC – South African health and nutrition claims; PAHO – Pan American Health Organization; CWO 2019 – Chile warning octagon 2019
