# Supplementary material for: Applying and comparing various nutrient profiling models against the packaged food supply in South Africa
Source: Public Health Nutr. 2022 Feb 16;25(8):2296–307. doi: 10.1017/S1368980022000374 (PMC9378746; doi:10.1017/S1368980022000374)
Supplement: Supplementary file 1 [file S1368980022000374sup.zip › S1368980022000374sup002.docx]

**Applying and comparing various nutrient profiling models against the packaged food supply in South Africa**

Tamryn Frank^1^, Shu Wen Ng^2^, Donna R. Miles^3^, Elizabeth C. Swart^4^

^1^ School of Public Health, University of the Western Cape Faculty of Community and Health Sciences, Cape Town, South Africa; tfrank@uwc.ac.za

^2^ Department of Nutrition, Gillings School of Global Public Health and the Carolina Population Center, The University of North Carolina Chapel Hill, United States of America; [shuwen@unc.edu](mailto:shuwen@unc.edu)

^3^ Carolina Population Center, The University of North Carolina Chapel Hill, United States of America; drmiles@unc.edu

^4^ Department of Dietetics and Nutrition, University of the Western Cape, South Africa, rswart@uwc.ac.za

**Corresponding author:** [Tamryn Frank; School of Public Health, University of the Western Cape; Private Bag X17, Bellville, South Africa, 7535; Researcher; tfrank@uwc.ac.za; +2784 782 9035]

**Short title:** Comparing_NPMs_against_SA_food_supply

**Keywords:** Nutrient profiling; South Africa; food policy; nutrients of concern; obesity

**Acknowledgements:** We wish to thank Barry Popkin for reviewing early versions of the manuscript, Jessica Ostrowski and Bridget Hollingsworth for exceptional research assistance and Alice Khan for superb data quality control. We also want to thank Lucrechia McAllen, Vashika Chibba, Thandokazi Mahuzi and Kholiswa Manisi, who conducted nutrition label data collection in 2018. For the software used to collect and enter the nutrition label data, we thank the National Center for Advancing Translational Sciences (NCATS), NIH UL1TR001111. Support from the DSI/NRF Center of Excellence in Food Security (UID91490) is also acknowledged.

**Financial Support: This research was funded by Bloomberg Philanthropies, with additional support from the US NIH grant number CPC P2C HD050924. Student scholarship was funded by International Research Development Canada (IDRC).**

**Conflict of Interest: The authors declare no conflict of interest. The funders had no role in the design of the study; in the collection, analyses, or interpretation of data; in the writing of the manuscript, or in the decision to publish the results.**

**Authorship: T.F., S.W.N, and E.C.S conceptualized planned the methodology for the manuscript; validation was done by T.F., D.R.M., S.W.N., and E.C.S.; T.F. and D.R.M. took care of data curation.; formal analysis, investigation and original draft preparation was carried out by T.F.; while review and editing was performed by T.F., S.W.N, D.R.M., E.C.S; visualization by T.F.; funding acquisition and resources were handled by S.W.N, and E.C.S; and E.C.S supervised the project.**

**Ethical Standards Disclosure:** This project was approved by the Human and Social Sciences Research Ethics committee HS19/6/3 of the University of the Western Cape, SA, and the Internal Review Board Reference 268261 of the University of North Carolina at Chapel Hill.
